# Vitamin K and childhood cancer: a report from the United Kingdom Childhood Cancer Study

**DOI:** 10.1038/sj.bjc.6601278

**Published:** 2003-09-30

**Authors:** N T Fear, E Roman, P Ansell, J Simpson, N Day, O B Eden

**Affiliations:** 1 Leukaemia Research Fund Epidemiology and Genetics Unit, University of Leeds, Leeds LS2 9JT, UK; 2 Strangeways Research Laboratory, The Institute of Public Health, University of Cambridge, Wort's Causeway, Cambridge CB1 8RN, UK; 3 Academic Unit of Paediatric Oncology, Christie Hospital and Central Manchester and Manchester Children's University Hospitals NHS Trusts, Wilmslow Road, Withington, Manchester M20 4BX, UK

**Keywords:** case–control study, childhood cancer, intramuscular administration, leukaemia, vitamin K

## Abstract

The relationship between neonatal vitamin K received by the intramuscular (i.m.) route and the development of leukaemia or other cancers was investigated as part of a national case–control study of childhood cancer, using data abstracted from obstetric and neonatal records. The analyses included 2530 children diagnosed with cancer before 15 years of age, 1174 of whom had leukaemia and 4487 control children without cancer. Overall, 39% of cases and 42% of controls had records of i.m. vitamin K administration, while 24% of cases and 22% of controls had no record of whether or not they had received vitamin K. Using subjects who received i.m. vitamin K as the baseline group, our analyses found no association between the administration of i.m. vitamin K and either leukaemia or other cancers as a group. We conclude that there is no convincing evidence that neonatal vitamin K administration, irrespective of the route by which it is given, influences the risk of children developing leukaemia or any other cancer.

Vitamin K given by the intramuscular (i.m.) route is known to be effective in preventing both early haemorrhagic disease in neonates and late-onset bleeding in older babies (Dam *et al*, 1952; McNinch and Tripp, 1991; Zipursky, 1999). The efficacy of oral vitamin K is less certain as there is a recognised problem of absorption in some babies, and additional difficulties in compliance with multiple-dose oral regimens (Zipursky, 1999; Wariyar *et al*, 2000; von Kries *et al*, 2003).

Concerns about the administration of i.m. vitamin K were raised by Golding *et al* (1992) in their report associating i.m., but not oral, vitamin K with childhood leukaemia. Since then, most research has failed to find any statistically significant associations (Ekelund *et al*, 1993; Klebanoff *et al*, 1993; Olsen *et al*, 1994; Ansell *et al*, 1996; von Kries *et al*, 1996; Roman *et al*, 1997; McKinney *et al*, 1998; Passmore *et al*, 1998a,1998b), although Parker *et al* (1998) argued that i.m. vitamin K increases the risk of acute lymphoblastic leukaemia (ALL) diagnosed between 12 and 71 months of age. However, a recently published pooled analysis of data from the six major case–control studies concluded that there was ‘no convincing evidence that i.m. vitamin K is associated with childhood leukaemia’, including ALL diagnosed between 12 and 71 months of age (Roman *et al*, 2002). Given the concerns associated with the administration of i.m. vitamin K, a wide variety of policies now operate throughout the United Kingdom (McNinch, 1997; Zipursky, 1999; Ansell *et al*, 2001).

The findings for neonatal vitamin K prophylaxis, assembled during the course of the large multicentre United Kingdom Childhood Cancer Study (UKCCS), are the focus of this report (UK Childhood Cancer Study Investigators, 2000).

## DATA AND METHODS

### Subjects

The United Kingdom Childhood Cancer Study (UKCCS) is a national population-based case–control study, full details of which are described elsewhere (UK Childhood Cancer Study Investigators, 2000). Briefly, children aged 0–14 years resident in the UK and diagnosed with pathologically confirmed malignancy between 1992 and 1996 were eligible for inclusion. For each case, two controls matched on sex, month and year of birth, and region of residence at diagnosis were randomly selected and recruited from population registers held by the (former) Family Health Service Authorities. At the time of the interview, parents were asked for consent to abstract their own and their child's medical records, including neonatal and obstetric notes. Information was systematically abstracted from obstetric and neonatal records by specially trained abstractors onto a previously validated, structured form designed to be applicable across hospitals and time periods. Data were abstracted on a wide variety of obstetric and neonatal events, including whether or not vitamin K was given and, if given, by which route.

Analyses were confined to England and North Wales, as data were not collected in South Wales and findings for Scotland have been previously published (McKinney *et al*, 1998,^1999^).

Of the 2692 cases and 4864 controls available for analyses, 162 cases (6%) and 377 controls (8%) were excluded–56 case children diagnosed under 3 months of age; 35 children with Down's syndrome; 168 who were the product of multiple pregnancies; and 280 who did not have a corresponding matched case or control (the majority were controls whose pseudodiagnosis age was less than 3 months–because their corresponding case was diagnosed under 3 months of age). After making these exclusions, 2530 cases and 4487 controls remained, 93% of the subjects whose obstetric notes were abstracted (Table 1

**Table 1:** Subjects excluded from the analysis by reason for exclusion and characteristics of those included

	**Cases**	**Controls**
Total available	2692 (100%)	4864 (100%)
*Excluded* (%)	162 (6%)^a^	377 (8%)
Diagnosed under 3 months of age	56	—
Down's syndrome	32	3
Multiple birth	60	108
Unmatched^b^	14	266
*Included* (%)	2530 (94%)	4487 (92%)

Total included	2530 (100%)	4487 (100%)
*Sex* (%)		
Males	1413 (56%)	2487 (55%)
Females	1117 (44%)	2000 (45%)

*Age at diagnosis* (%)		
<1 year	189 (7%)	351 (8%)
1 year	254 (10%)	473 (11%)
2–5 years	1069 (42%)	1926 (43%)
6+ years	1018 (40%)	1737 (39%)

a Three cases diagnosed under 3 months of age also had Down's syndrome, and two cases with Down's syndrome were also part of a multiple birth.

b Cases or controls who did not have a corresponding matched case or control after the exclusions had been made.

). Analyses were carried out for all cancers combined (2530 cases), and separately for all leukaemias combined (1174 cases), acute lymphoblastic leukaemia (ALL, 1001 cases) and all other cancers except leukaemia (1356 cases).

The odds ratios (ORs), 95% confidence intervals (CIs) and two-sided *P*-values were estimated using unconditional logistic regression (Breslow and Day, 1980) with adjustment for the region of residence at diagnosis (eight in total), sex and age at diagnosis (in single years). To maximise the robustness of the analyses, the total pool of controls was the comparator for each diagnostic group, an approach adopted in other UKCCS analyses (e.g. Beral *et al*, 2001). Statistical analyses were conducted using STATA (version 7.0) (StataCorp, 2001).

## RESULTS

Table 2

**Table 2:** Distribution of cases and controls, associated odds ratios (OR) and 95% confidence intervals (CI) by vitamin K status

		**Leukaemia**			
**Vitamin K status**	**All cancers (*n*=2530)**	**Total (*n*=1174)**	**ALL**^a^ **(*n*=1001)**	**Other cancers**^b^ **(*n*=1356)**	**Controls (*n*=4487)**
*Number* (%)					
Intramuscular	991 (39%)	456 (39%)	394 (39%)	535 (39%)	1868 (42%)
Oral	376 (15%)	181 (15%)	154 (15%)	195 (14%)	663 (15%)
Given but route unknown	455 (18%)	233 (20%)	199 (20%)	222 (16%)	815 (18%)
Not given	106 (4%)	39 (3%)	30 (3%)	67 (5%)	162 (4%)
No record	602 (24%)	265 (23%)	224 (22%)	337 (25%)	979 (22%)

*OR (95% CI)*^c^					
Intramuscular	1.0	1.0	1.0	1.0	—
Oral	1.09 (0.94–1.27)	1.13 (0.92–1.38)	1.11 (0.90–1.38)	1.06 (0.87–1.29)	—
Given but route unknown	1.05 (0.91–1.22)	1.19 (0.99–1.43)	1.16 (0.95–1.41)	0.95 (0.79–1.14)	—
Not given	1.24 (0.95–1.61)	1.07 (0.74–1.55)	0.98 (0.65–1.49)	1.39 (1.03–1.90)*	—
No record	1.15 (1.01–1.31)*	1.18 (0.99–1.41)	1.19 (0.99–1.44)	1.12 (0.95–1.32)	—

a ALL=Acute lymphoblastic leukaemia.

b Cancers other than leukaemia.

c Calculated using logistic regression with adjustment for age at diagnosis (single years), sex and region of residence.

* *P*<0.05.

shows the distribution of cases and controls, associated ORs and 95% CIs by vitamin K status. Overall, 39% of cases and 42% of controls were recorded as having received i.m. vitamin K shortly after birth, with 18% of both cases and controls recorded as having received vitamin K without the route being recorded, and 24% of cases and 22% of controls having no information on whether vitamin K was given (or not given) written in their notes. There are no notable differences in the distribution of vitamin K status by case–control status.

Using the large group of subjects known to have received i.m. vitamin K as the baseline, no statistically significantly increased risks for leukaemia are apparent (Table 2). For all cancers combined, however, the OR was 1.15 (95% CI=1.01–1.31) when no written record of administration was found, and for cancers other than leukaemia the OR was 1.39 (1.03–1.90), when vitamin K was recorded as not given. Children diagnosed with leukaemia aged between 12 and 71 months were no more or less likely to have received i.m. vitamin K than children who were not diagnosed with cancer (Table 3

**Table 3:** Distribution of cases and controls, associated odds ratios (OR) and 95% confidence intervals (CI) by vitamin K status for subjects aged 12–71 months

		**Leukaemia**			
**Vitamin K status**	**All cancers**	**Total**	**ALL**^a^	**Other cancers^**b**^**	**Controls^b^**
*Number* (%)					
Intramuscular	531 (40%)	294 (40%)	265 (40%)	237 (40%)	1037 (43%)
Oral	238 (18%)	135 (18%)	119 (18%)	103 (17%)	444 (19%)
Given but route unknown	266 (20%)	148 (20%)	134 (20%)	118 (16%)	461 (18%)
Not given	39 (3%)	15 (2%)	12 (2%)	24 (4%)	74 (3%)
No record	249 (19%)	140 (19%)	125 (19%)	109 (18%)	383 (16%)

*OR* (95% CI)^c^					
Intramuscular	1.0	1.0	1.0	1.0	—
Oral	1.06 (0.88–1.29)	1.16 (0.88–1.42)	1.10 (0.86–1.41)	1.01 (0.78–1.32)	—
Given but route unknown	1.13 (0.93–1.36)	1.12 (0.89–1.42)	1.13 (0.88–1.44)	1.14 (0.88–1.47)	—
Not given	1.04 (0.69–1.56)	0.73 (0.41–1.30)	0.66 (0.35–1.24)	1.43 (0.87–2.32)	—
No record	1.28 (1.06–1.56)*	1.29 (1.02–1.64)*	1.28 (1.00–1.64)*	1.27 (0.98–1.65)	—

a ALL=Acute lymphoblastic leukaemia.

b Cancers other than leukaemia.

c Calculated using logistic regression with adjustment for age at diagnosis (single years), sex and region of residence.

**Figure 1:**
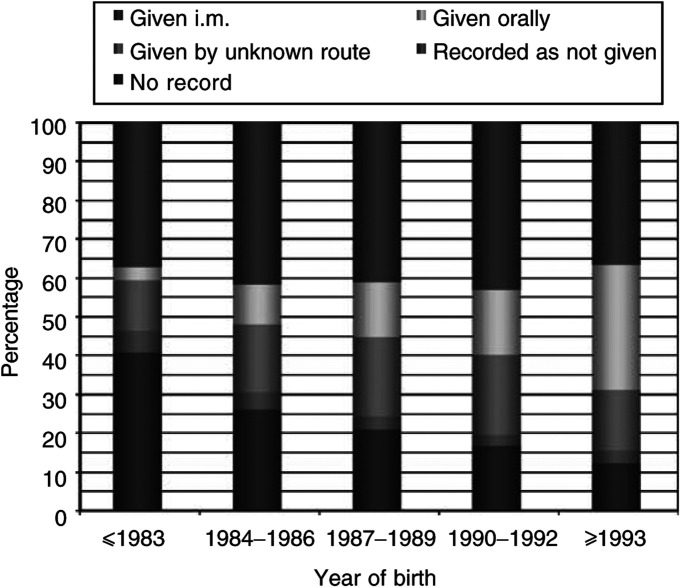
Recording of vitamin K administration by year birth.

* *P*<0.05.

). The proportion of subjects with no recorded information is, however, generally higher in Table 2 (24% of cases and 22% of controls) than in Table 3 (19% of cases and 16% of controls). This reflects the fact that the children included in the latter table were, on an average, born more recently (data not shown)–hospital recording practices have improved over time and babies are now also more likely to receive vitamin K, particularly by the oral route (Figure 1

).

All analyses were repeated with adjustment for low birth weight (<2501 g, ⩾2501 g), mode of delivery (normal vaginal, caesarean section, other), admission to special care baby unit (no+unknown, yes) and year of birth, but the findings were unchanged (data not shown).

## DISCUSSION

Our analyses, based on neonatal and obstetric records collected from maternity units across England during the course of a large population-based case–control study (UK Childhood Cancer Study Investigators, 2000), found no evidence of an association between i.m. vitamin K and childhood cancer in general, or leukaemia in particular. With 2530 cases of cancer–over a thousand of which were leukaemias–and 4487 controls, this is the most comprehensive study of its kind to have been reported on this topic.

Although previous investigators tended to follow Golding *et al*'s (1992) example and compared children with a written or imputed record of having received i.m. vitamin K with those who received it orally or not at all, we have not done so. Our design and large numbers permitted a comparison between five groups, those with:

1. a written record of having received vitamin K by the i.m. route,
2. a written record of having received vitamin K orally,
3. a written record of having received vitamin K, but by an unknown route,
4. a written record stating that they had not received vitamin K,
5. no written information (given or not given) regarding vitamin K administration.

Overall, 39% of cases and 42% of controls had a written record of i.m. vitamin K administration, while 24% of cases and 22% of controls had no record of whether or not they had received vitamin K. Using subjects who received i.m. vitamin K as the baseline, no association between i.m. vitamin K and either leukaemia or any other cancer was found. For cancers other than leukaemia, however, the OR was 1.39 (95% CI=1.03–1.90) when vitamin K was recorded as not given and for all cancers combined it was 1.15 (1.01–1.31), when no written record of administration was found. The increase in cancer risk associated with having no written vitamin K record became more pronounced when the analysis was restricted to children diagnosed between 12 and 71 months of age: the ORs for all cancers, ALL and cancers other than leukaemia being 1.28(1.06–1.56), 1.28(1.00–1.64) and 1.27(0.98–1.65), respectively. The generalised nature of this effect, coupled with the fact that cases born in smaller maternity units were more likely than cases born in larger units to have no written record of administration (data not shown), suggests that either chance or bias could have had a role to play in this result.

The lack of an association reported here between childhood leukaemia and the i.m. administration of vitamin K accords with the majority of individual studies to have reported on this topic (Ekelund *et al*, 1993; Klebanoff *et al*, 1993; Olsen *et al*, 1994; Ansell *et al*, 1996; von Kries *et al*, 1996; Roman *et al*, 1997; McKinney *et al*, 1998; Passmore *et al*, 1998a,1998b) and with the results of an individual record-based pooled analysis of the six major case–control studies (Roman *et al*, 2002). The one exception is a study carried out in the former Northern Health region of England which, although it found no association for all childhood ALL (OR=1.20, 95% CI=0.75–1.92, based on 207 cases), reported a statistically significantly raised OR for ALL diagnosed between 12 and 71 months of age (OR=1.79, 95% CI=1.02–3.15, based on 144 cases) (Parker *et al*, 1998). To investigate this further, a meta-analysis of the results published by Roman *et al* (2002) and the results reported here has been undertaken. In order to do this, the analyses reported here were repeated comparing oral+given unknown route+not given+no record (baseline group) with i.m. vitamin K. No associations were observed between i.m. vitamin K and any diagnostic group, with the pooled OR for ALL diagnosed between 12 and 71 months being: OR=0.98, 95% CI=0.79–1.22 (based on 1201 cases). In light of all the available evidence, the most likely explanation for the finding of Parker *et al* (1998) would seem to be chance.

Accordingly, we conclude that there is no convincing evidence that neonatal vitamin K administration, irrespective of the route by which it is given, influences the risk of children developing leukaemia or any other cancer.

## Appendix

*UKCCS Investigators*: KK Cheng^*^ and E Gilman, Central region; NE Day^*^, J Skinner and D Williams, East Anglia region; R Cartwright^*^ and A Craft^*^, Northeast region; JM Birch^*^ and OB Eden^*^, North west region; PA McKinney^*^, Scotland; J Deacon and J Peto^*^, Southeast region; V Beral^*^ and E Roman^*^, South Midlands region; P Elwood^*^, South Wales region; FE Alexander^*^ and M Mott, Southwest region; CED Chilvers^*^ and K Muir, Trent region; P Ansell, NT Fear, E Roman^*^ and G Simpson, Leukaemia Research Fund Data Management Processing Group; R Doll^*^, Epidemiological Studies Unit, University of Oxford, Oxford; CM Taylor^*^, Immunogenetics Laboratory, University of Manchester, Manchester; M Greaves^*^, Leukaemia Research Fund Centre, Institute of Cancer Research.

*Management Committee*: ^*^UKCCS Investigators and D Goodhead, Radiation and Genome Stability Unit, Medical Research Council, Harwell; FA Fry, National Radiological Protection Board; G Adams, UK Coordinating Committee for Cancer Research.
